# Charge distribution guided by grain crystallographic orientations in polycrystalline battery materials

**DOI:** 10.1038/s41467-019-13884-x

**Published:** 2020-01-08

**Authors:** Zhengrui Xu, Zhisen Jiang, Chunguang Kuai, Rong Xu, Changdong Qin, Yan Zhang, Muhammad Mominur Rahman, Chenxi Wei, Dennis Nordlund, Cheng-Jun Sun, Xianghui Xiao, Xi-Wen Du, Kejie Zhao, Pengfei Yan, Yijin Liu, Feng Lin

**Affiliations:** 1 Department of Chemistry, Virginia Tech, Blacksburg, VA 24061 USA; 2 Stanford Synchrotron Radiation Lightsource, SLAC National Accelerator Laboratory, Menlo Park, CA 94025 USA; 3 Institute of New-Energy Materials, School of Materials Science and Engineering, Tianjin University, Tianjin, 300072 China; 4 School of Mechanical Engineering, Purdue University, West Lafayette, IN 47907 USA; 5 Beijing Key Laboratory of Microstructure and Properties of Solids, Beijing University of Technology, Beijing, 100124 China; 6 Advanced Photon Source, Argonne National Laboratory, Argonne, IL 60439 USA; 7 National Synchrotron Light Source II, Brookhaven National Laboratory, Upton, NY 11973 USA

**Keywords:** Chemistry, Energy science and technology, Materials science

## Abstract

Architecting grain crystallographic orientation can modulate charge distribution and chemomechanical properties for enhancing the performance of polycrystalline battery materials. However, probing the interplay between charge distribution, grain crystallographic orientation, and performance remains a daunting challenge. Herein, we elucidate the spatially resolved charge distribution in lithium layered oxides with different grain crystallographic arrangements and establish a model to quantify their charge distributions. While the holistic “surface-to-bulk” charge distribution prevails in polycrystalline particles, the crystallographic orientation-guided redox reaction governs the charge distribution in the local charged nanodomains. Compared to the randomly oriented grains, the radially aligned grains exhibit a lower cell polarization and higher capacity retention upon battery cycling. The radially aligned grains create less tortuous lithium ion pathways, thus improving the charge homogeneity as statistically quantified from over 20 million nanodomains in polycrystalline particles. This study provides an improved understanding of the charge distribution and chemomechanical properties of polycrystalline battery materials.

## Abstract (web summary)

The authors here report on the influence of grain orientation on the charge distribution in polycrystalline materials for batteries. The quantitative characterization provides mechanistic insight into the way the grain orientation can be engineered to mitigate the charge heterogeneity.

## Introduction

Solid state redox reactions are ubiquitous during ion reactions in batteries^1^ and catalysts^2^. Understanding how redox reactions propagate can inform designing internal microstructures such that redox reactions can be fully accessed to deliver the desired functionalities. The propagation of redox reactions governs the electrochemical properties of battery materials and their critical performance metrics in practical cells^3–5^. The recent research progress, especially aided by advanced analytical techniques^6^, has revealed that incomplete and heterogeneous redox reactions prevail in many electrode materials, such as olivine phosphates^7–14^, layered oxides^15–20^, spinel oxides^21,22^, and conversion materials^23,24^. Advanced high-capacity cathode materials for lithium (Li) ion and sodium (Na) ion batteries are mostly polycrystalline materials that exhibit complex charge distribution (the valence state distribution of the redox-active cations) due to the presence of numerous constituting grains and grain boundaries. The redox reactions in individual grains typically do not proceed concurrently due to their distinct geometric locations in polycrystalline particles. As a result, these unsynchronized local redox events collectively induce heterogeneous and anisotropic charge distribution, building up intergranular and intragranular stress^25^. Therefore, these polycrystalline materials may exhibit weak mechanical stability, leading to undesired chemomechanical breakdown during battery cycling^26–29^.

High-nickel, low-cobalt layered oxides show great promise to reduce or even eliminate the use of high-cost, toxic, and socially controversial cobalt in Li ion batteries. However, these materials undergo many phase transformations above 4.0 V vs Li^+^/Li^30^, which create large internal stress along grain boundaries and engender microcrack formation^25,31–33^. To fully unlock the high-energy advantage of these nickel-rich layered oxides, methods must be established to guide the redox reaction and to enhance the chemomechanical stability of these polycrystalline materials. Recent studies have discovered that engineering the grain crystallographic orientation could modulate the ion transport pathway in polycrystalline particles to enable rapid ion reactions^4,5,34–37^. Furthermore, these studies have concluded that the improved battery performance was attributed to the enhanced chemomechanical properties. Several studies have attempted to investigate the relationship between the compositional^38–40^, morphological^26,41,42^, and charge complexities^15,43^ and the battery performance of polycrystalline battery particles. However, there is a lack of a holistic study to spatially and quantitatively elucidate the interplay between grain crystallographic orientation, charge distribution, and battery performance.

Herein, we report the distinct charge distributions (Ni valence state distribution) in two compositionally similar, yet, crystallographically dissimilar (randomly oriented grains vs radially aligned grains) polycrystalline nickel-rich layered oxides. Under the stringently controlled electrochemical protocol, these two layered oxides not only deliver nearly identical initial capacity and voltage profile but also exhibit similar structural and chemical characteristics. Overall, both materials display a “surface-to-bulk” charge distribution modality. However, the three-dimensional charge mapping, covering over 20 million nanodomains, reveals that the internal charge distribution is guided by the crystallographic orientation of the constituent grains. Our mathematical model elucidates that the radially aligned grains, with continuous and radially distributed Li ion pathways, favor the overall charge homogeneity. These results provide fundamental insights into tuning grain crystallographic orientation to improve performance robustness in practical batteries.

## Results

### Li layered oxides of different crystallographic orientations

**Fig. 1:**
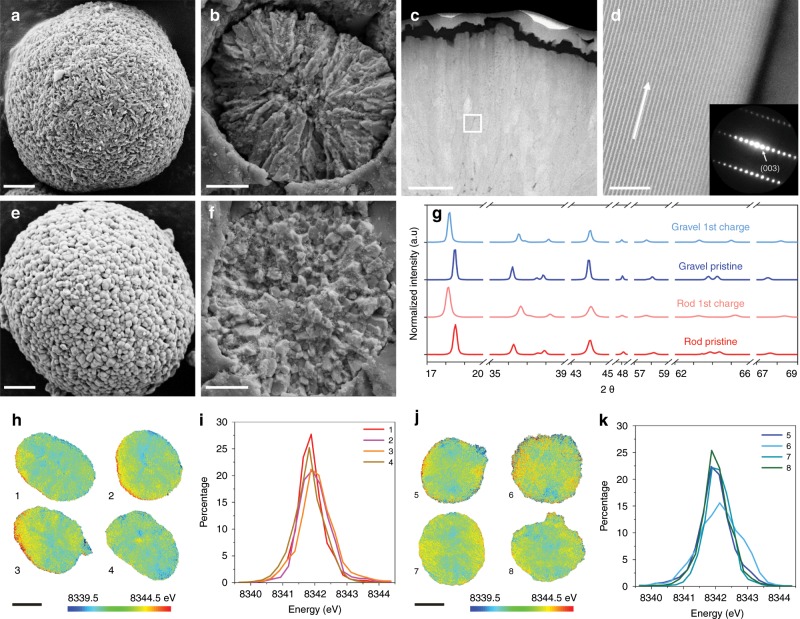
Morphology, crystal structure, and charge distribution of the rod- and gravel-NMCs. **a** Overview and **b** interior SEM images of rod-NMC. **c** Cross-section and **d** high resolution STEM images of rod-NMC grains. Panel (**d**) corresponds to the square in (**c**). The nano-beam diffraction pattern of the fringes in (**d**) is included as an inset in (**d**). **e** Overview and **f** interior SEM images of gravel-NMC. **g** XRD patterns of rod- and gravel-NMCs in the pristine and the first charged state (Cut-off voltage: 4.5 V vs Li^+^/Li; Current: C/5 (1C = 200 mA g^−1^); Capacity: ~210 mAh g^−1^). **h** 2D TXM images and **i** Ni K-edge energy histograms of four rod-NMC secondary particles in the pristine state. **j** 2D TXM images and **k** Ni K-edge energy histograms of four gravel-NMC secondary particles in the pristine state. The scale bars in (**a**), (**b**), (**c**), (**d**), (**e**), (**f**), (**h**), and (**j**) are 2 μm, 2 μm, 1 μm, 5 nm, 2 μm, 2 μm, 5 μm, and 5 μm, respectively. The Ni K-edge energy in (**h**) and (**j**) are color-coded, where blue and red stand for low edge energy (low valence state) and high edge energy (high valence state), respectively.

Two nickel-rich Li layered oxides (LiNi_x_Mn_y_Co_1-x-y_O_2_, NMC) with similar redox-active nickel (Ni) content yet different grain crystallographic orientations, named as rod-NMC (with radially aligned grains) and gravel-NMC (with randomly oriented grains), were adopted as the model systems. The energy-dispersive X-ray spectroscopy (EDS, Supplementary Fig. 1) and inductively coupled plasma-mass spectrometry (ICP-MS, Supplementary Table 1) both confirm that the redox-active Ni content among the three transition metals is similar for the rod-NMC (~82%) and gravel-NMC (~80%). These two NMCs are of distinctive morphologies and Li ion pathways, as attested by the scanning electron microscopy (SEM) and scanning transmission electron microscopy high-angle annular dark-field (STEM-HAADF) imaging and nano-beam diffraction (NBD) results. For the rod-NMC, the spherical secondary particles (Fig. 1a) are formed by radially packed rod-shaped grains (Fig. 1b). The radially packed morphology is further demonstrated in Supplementary Fig. 2, in which multiple methods were used to expose the interior of many rod-NMC secondary particles. The Li channels, as illustrated by the arrows in Fig. 1d and Supplementary Fig. 3, are along the radial direction in the rod-shaped grains, indicating that Li ions can transport readily from the bulk of the secondary particle to the surface in the radial direction^4,5,34^. In contrast, the spherical secondary particles of the gravel-NMC (Fig. 1e) mainly comprise randomly packed gravel-shaped grains (Fig. 1f, Supplementary Fig. 4). The random packing of the grains implies that the Li ion pathways in gravel-NMC are random and likely tortuous. Next, we performed comprehensive multiscale characterization to ensure that we had a structurally and chemically controlled model system to investigate how the crystallographic orientation guides the charge distribution.

**Fig. 2:**
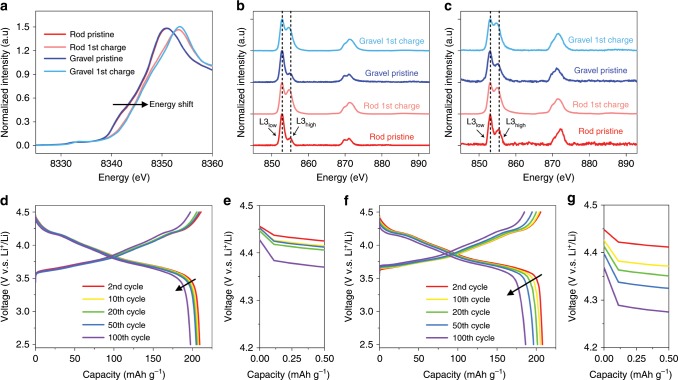
Electronic structure and electrochemical performance of the rod- and gravel-NMCs. **a** Ni K-edge XAS, **b** Ni L-edge XAS (TEY mode), and **c** Ni L-edge (FY mode) of the rod- and gravel-NMCs in the pristine and the first charged state. **d** Charge-discharge profile and **e** voltage drop of rod-NMC at 2.5–4.5 V for 100 cycles at C/5 (1C = 200 mA g^−1^). **f** Charge-discharge profile and **g** voltage drop of gravel-NMC at 2.5–4.5 V for 100 cycles at C/5 (1C = 200 mA g^−1^).

Although the two NMCs have different morphologies and crystallographic orientations, they do have similar crystal structure in the pristine and first charged state (Supplementary Fig. 5), respectively. Synchrotron X-ray diffraction (XRD) reveals that all NMCs have the R$\bar 3$m structure and the peak positions are similar for the two NMCs in the pristine and first charged state, respectively (Fig. 1g, Supplementary Fig. 6). The powder XRD Rietveld refinement of the two NMCs confirms that their lattice parameters are similar in the pristine state (Supplementary Fig. 6). In addition, the (003)/(104) peak ratio is 1.337 and 1.355 for the rod-NMC and gravel-NMC, respectively. The difference is minor, suggesting the similar cation mixing in these pristine samples. The peak shifts in the charged state are also almost identical for both NMCs, which is consistent with the XANES results (Fig. 2a, Supplementary Table 2) and shows that the same state of charge has reached for the two samples. To further compare the two NMCs, their charge distributions in the pristine state were investigated via the full field transmission X-ray microscopy (TXM). TXM can detect the local valence state by measuring the absorption spectrum of a specific element with a nominal spatial resolution of ~30 nm. Ni is the primary charge compensating element in nickel-rich layered oxides at 2.5–4.5 V, the redox reaction propagation in the NMCs can thus be investigated through quantifying the evolution of Ni valence states. Two-dimensional (2D) TXM was performed on multiple pristine secondary particles, and the results show that the pristine rod-NMC (Fig. 1h–i) and gravel-NMC (Fig. 1j–k) have similar charge distribution. The Ni valence state histograms for the two NMCs are also similar (Fig. 1i, k). The difference of the average Ni K-edge absorption energy for the rod- and gravel-NMC is only about 0.12 eV, which is much smaller than the energy resolution of TXM (~0.8 eV) and demonstrates that the two NMCs have similar charge distribution in the pristine state^18,43^.

In addition to the charge distribution in pristine state, the global bulk and surface charge states were investigated using hard and soft X-ray absorption spectroscopy (XAS) techniques, respectively. To be specific, the hard XAS can detect the bulk Ni valence state by detecting the Ni K-edge absorption energy, while soft XAS was used to measure the surface Ni valence state by measuring the Ni L-edge absorption spectroscopic fingerprint^6^. The hard XAS spectra (Fig. 2a, Supplementary Table 2) show that the Ni has almost identical valence state in the pristine state, and after charging to 4.5 V, Ni gets oxidized to a similar extent for both NMCs. The soft XAS spectra indicate that the surface Ni valence states are also similar for both NMCs. Two signal acquisition modes were used in this work: total electron yield (TEY) mode with a probing depth of 5–10 nm and fluorescence yield (FY) mode with a probing depth around 50 nm^44^. Two L3 peaks can be obtained in the Ni soft XAS spectrum and the L3_high_/L3_low_ ratios (Supplementary Table 3) can be used to monitor the surface Ni valence state: a larger L3_high_/L3_low_ value means a higher valence state, and vice versa^45^. In the TEY mode (Fig. 2b), the rod- and gravel-NMCs have similar Ni valence state in their pristine state. After the first charge, the L3_high_/L3_low_ peak ratio increase to a similar level, which indicates the surface Ni get oxidized to a similar extent. Similarly, under the FY mode (Fig. 2c), where signals are mainly from the sub-surface, the L3_high_/L3_low_ ratios are also comparable in the pristine and the first charged state for both NMCs. In summary, the hard and soft XAS results show that the overall bulk and surface Ni valence states are similar for the two NMCs in their pristine and first charged state, respectively.

The rod-NMC (Fig. 2d) has a 209.3 mAh g^−1^ discharge capacity at the second discharge, and, after 100 cycles, the cell remains a capacity of 197.4 mAh g^−1^ (94.3% retention). Besides, the rod-NMC has negligible voltage drop between the upper cut-off voltage and the onset of the discharge curve: the onset discharge voltage is 4.46 V for the second cycle and 4.43 V for the 100th cycle (Fig. 2e). The gravel-NMC (Fig. 2f) has a comparable initial capacity (207.8 mAh g^−1^ for the second discharge), but the capacity retention is noticeably lower after 100 cycles (186.3 mAh g^−1^, 89.7% retention). The voltage drop of gravel-NMC is also more significant (Fig. 2g). The voltage at the onset of the second discharge is 4.45 V, which is comparable to that of rod-NMC. However, with much faster polarization induced voltage drop during the cycling process, the onset discharge voltage is only 4.37 V at the 100th discharge for the gravel-NMC. We performed the performance measurements for more than three times and the averaged results, shown in Supplementary Fig. 7, clearly show that the rod-NMC exhibits more robust battery performance.

### Charge distributions in the charged state

**Fig. 3:**
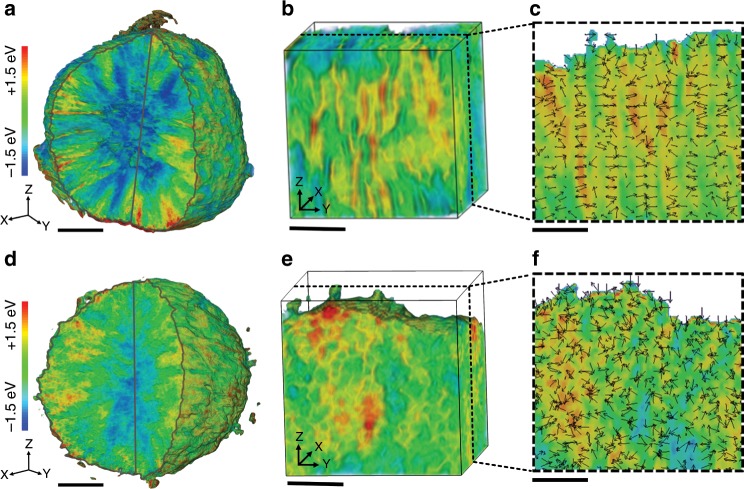
Charge distributions in the first charged state. **a** 3D Ni valence state distribution, **b** representative region of the 3D Ni valence state distribution, and **c** 2D nanodomain valence gradient of the rod-NMC. **d** 3D Ni valence state distribution, **e** representative region of the 3D Ni valence state distribution, and **f** 2D nanodomain valence gradient of the gravel-NMC. The nanodomain valence gradient vectors are represented by the black arrows, where the vector direction and magnitude are represented by the arrow direction and arrow length, respectively. The scale bars in (**a**) and (**d**) are 3 μm, and the scale bars in (**b**–**c**) and (**e**–**f**) are 1 μm. The Ni K-edge absorption energies are color-coded, in which blue stands for lower edge energy and red means higher edge energy.

As discussed earlier, charge heterogeneity has been reported in many battery materials. Recently, Chueh and coworkers reported that the charge heterogeneity is persistent in Li_x_Ni_1/3_Mn_1/3_Co_1/3_O_2_ (NMC333) even after prolonged relaxation (more than 170 h)^46^. Moreover, another study suggests that charge heterogeneity in battery materials is likely less rate-dependent. The authors observed significant extent of charge heterogeneity in the slowly charged (C/10) Li_x_Ni_0.6_Mn_0.2_Co_0.2_O_2_ (NMC622)^18^. Here we conduct the charge distribution mapping for nickel-rich NMC in the charged state after a slow charging process (C/10), and we also observe a large degree of charge heterogeneity (Supplementary Fig. 8). Such a prevailing charging heterogeneity phenomenon motivates us to perform a quantitative study about the effect of grain orientation on the charge heterogeneity. In our current study, although the differences in the Ni content, lattice structure and global Ni valence state are negligible between the rod- and gravel-NMCs, the rod-NMC demonstrates improved capacity retention and suppressed polarization. Therefore, we conjecture that the different grain crystallographic orientations play a major role in affecting the redox reaction pathway and charge distribution in the secondary particles, which, in turn, influence their electrochemical performance. Herein, the underlying mechanism was investigated using three-dimensional (3D) TXM, which can probe the local Ni valence state with a 30 × 30 × 30 nm^3^ nanodomain resolution^47,48^. Thin film electrodes (~40 μm) were used in this work and the depth-resolved confocal hard XAS (Supplementary Fig. 9) shows that, at the charged state, the local state-of-charge within the thin film electrodes is independent of the depth. Such electrode scale state-of-charge homogeneity promotes the representativeness of the particle level studies. The overall charge distributions within selected particles of the rod- and gravel-NMCs are shown in Fig. 3a, d. Because of the favorable electrical and ionic conductivity near the particle surface, the surface is more oxidized than the bulk in both NMCs, resembling the “surface-to-bulk” model at the secondary particle level (Fig. 3a, d, and Supplementary Fig. 10). However, the spatial pattern of the varying nickel valence state is different, as shown in Fig. 3b, e, for rod- and gravel-NMC, respectively. The 2D nanodomain valence gradient vectors, which are defined as the local variation of the absorption edge energy in two dimension, are mostly parallel to each other for rod-NMC (Fig. 3c) and are random for gravel-NMC (Fig. 3f). Such Ni valence state distribution and 2D nanodomain valence gradient are attributed to the different redox reaction behaviors that are likely guided by the grain crystallographic orientation. The high valence Ni domains are radially oriented in the rod-NMC, in good alignment with the grain crystallographic arrangement. Moreover, the high valence Ni domains appear to be interconnected in 3D, and their size is comparable to that of the single crystalline grains (Fig. 1b, Supplementary Fig. 2).

Based on the experimentally observed charge distribution, here we propose a hypothesis regarding the reaction pathways in the rod-NMC. The delithiation reaction nucleates from single grain surface and propagates to the bulk of each single grain. The redox reaction, on the other hand, can be illustrated using the valence gradient (Fig. 3c)^49^, which is mostly populated in the tangential orientation for the rod-NMC. After the initiation of delithiation at the single crystal surface, the increased d_(003)_ distance facilitates Li ion transport, which further promotes the delithiation at the surface of single crystal surface along the (003) plane. Combining the TEM (Fig. 1c–d, Supplementary Fig. 3) and 3D TXM results (Fig. 3a–c), we propose that the grain crystallographic orientations can guide the reaction pathway in the polycrystalline NMC particles and, therefore, determine the charge distribution. The alignment of Li ion pathways in the rod-NMC allows for rapid Li ion transport from the surface to the bulk of NMC secondary particles, which potentially contributes to the better performance at a higher charging/discharging rate (Supplementary Fig. 7)^4,5,34–37^. For the gravel-NMC, the delithiation also initiates from the surface, as attested by the distribution and dimension of the high valence Ni domains (Fig. 3d–f, Fig. 1f, Supplementary Fig. 4). However, those high valence Ni domains in gravel-MNC do not exhibit clear radial distribution, due to the fact that the gravel-shaped grains are randomly packed and, thus, the Li ion diffusion pathway is tortuous.

### Quantitative analysis of charge distributions

**Fig. 4:**
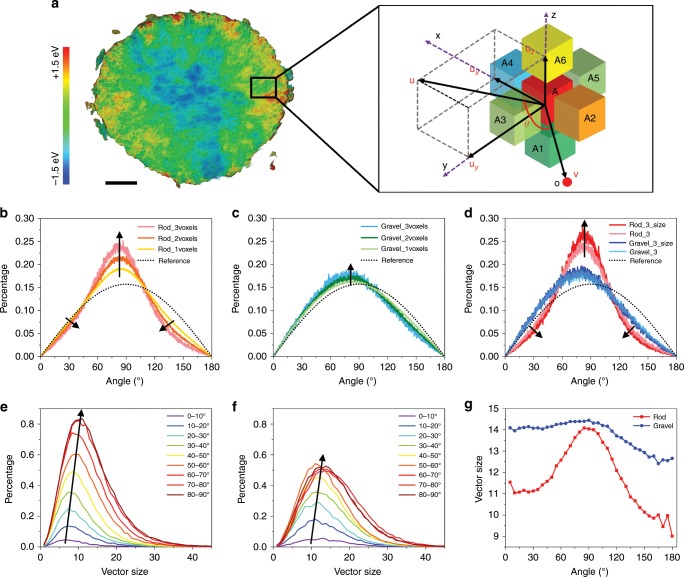
Quantitative analysis of the charge distributions. **a** Schematic illustration of the valence gradient model used to analyze the 3D distribution of Ni valence states. The left side is a cross-section image of the 3D TXM, in which the relative local Ni K-edge absorption energy are color-coded to illustrate the spatial variation of the Ni valence state. The vector angle *θ* distributions of **b** rod-NMC and **c** gravel-NMC as a function of voxel size. **d** The vector angle *θ* distributions of rod- and gravel-NMCs before and after considering the vector size. Rod_3 and Gravel_3 in (**d**) correspond to the Rod_3voxels and Gravel_3voxels in (**b**) and (**c**), respectively. Rod_3_size and Gravel_3_size in (**d**) are the results after considering the vector sizes. The dashed black lines in (**b**–**d**) represent the reference data as explained in Supplementary Fig. 13. The valence gradient vector size distributions in different *θ* ranges of **e** rod-NMC and **f** gravel-NMC. **g** The average valence gradient vector size as a function of *θ* where 0°–180° were divided to 36 intervals. The scale bar in (**a**) is 2 μm.

The 3D TXM mapping provides qualitative understanding about the redox reaction and charge distribution mechanisms. Here a mathematic model is developed to quantitatively investigate the effect of the grain crystallographic orientation on charge distribution and charge heterogeneity (overall procedures are shown in Supplementary Fig. 11). In our TXM result, each secondary NMC particle is spatially resolved by over 20 million unique voxels of ~30 × 30 × 30 nm^3^. While offering a great amount of structural and chemical details, such datasets provide an opportunity for a statistical analysis of the relationship between neighboring voxels. A valence gradient based model was developed to investigate the charge distribution in this work. As shown in Fig. 4a: each voxel (A in Fig. 4a) has six nearest neighboring voxels (A1, A2, A3, A4, A5, and A6 in Fig. 4a) and each voxel has its own Ni valence state, which is determined by the local Ni K-edge absorption energy. In each direction (*x*, *y*, and *z*), there is a partial valence gradient vector (**u**_***x***_**, u**_***y***_**, u**_***z***_), which is defined as:

1 $${\mathbf{u}}_{\mathbf{x}} = \frac{{{\mathrm{\Delta }}{\mathbf{E}}_{\boldsymbol{x}}}}{{{\mathrm{\Delta }}d_x}}$$

2 $${\mathbf{u}}_{\mathbf{y}} = \frac{{{\mathrm{\Delta }}{\mathbf{E}}_{\boldsymbol{y}}}}{{{\mathrm{\Delta }}d_y}}$$

3 $${\mathbf{u}}_{\mathbf{z}} = \frac{{{\mathrm{\Delta }}{\mathbf{E}}_{\boldsymbol{z}}}}{{{\mathrm{\Delta }}d_z}}$$

Δ**E**_***x***_, Δ**E**_***y***_, and Δ**E**_***z***_ are the local variation of the absorption edge energy in *x*, *y*, and z directions, respectively. The total valence gradient vector (**u**) is the vector sum of the three partial valence gradient vectors:

4 $${\mathbf{u}} = {\mathbf{u}}_x + {\mathbf{u}}_y + {\mathbf{u}}_z$$

For each voxel, connecting it back to the gravity center of the polycrystalline particle (point O in Fig. 4a) gives us another vector **v**. The angle (*θ*) between **u** and **v** can be calculated as:

5 $$\theta = {\mathrm{arc}}\;{\mathrm{cos}}\left( {\frac{{{\mathbf{u}} \cdot {\mathbf{v}}}}{{\left| {\mathbf{u}} \right|\left| {\mathbf{v}} \right|}}} \right)$$

The recovered *θ* value in this model is related to the local gradient of the Ni valence states, while the size of **u** is proportional to the charge difference (heterogeneity) between the neighboring voxels. To buildup the model, we first focus on the vector angles (*θ*) and the amplitude of the vectors (**u**) is disregarded for now. Moreover, merging the voxels (i.e., artificially reducing the spatial resolution) can help us to amplify the contribution from regions in the vicinity of grain boundaries (Supplementary Fig. 12). In this model, 2 or 3 voxels were combined in the *x*, *y*, and *z* directions, which corresponds to averaging the Ni K-edge energy of 8 (2 × 2 × 2) or 27 (3 × 3 × 3) voxels, respectively. The probability distribution of the *θ* value for the two NMCs are shown in Fig. 4b, c, respectively. The *θ* value distribution all have a volcano shape, but with different sharpness. The volcano shape *θ* distribution is intrinsic to our model (Supplementary Fig. 13). Under the assumption of fully random charge distribution, the distribution of vectors is proportional to sin *θ*, which is plotted as our baseline reference in Fig. 4b–d. Comparing with the reference, the rod-NMC (Fig. 4b) has more valence gradient vectors with *θ* near 90°, and the peak sharpness increases dramatically as the voxel size increases. This sharp volcano shape is a fingerprint for the radial distribution of the Ni valence state. In contrast, the gravel-NMC (Fig. 4c) angle distribution is similar to the reference and the change is minor when the effective voxel size is tuned, suggesting that the gravel-NMC has relatively random charge distribution. In both cases (Fig. 4b, c), the peak center is not exactly at 90°, which can be explained by the fact that the secondary particles are not perfect spheres (Fig. 3a, d, Supplementary Figs. 1 and 3).

In the next step, the vector amplitude was taken into consideration for the 3-voxels binning data. Every vector **u** was divided into multiple unit vectors in the same direction based on the corresponding amplitude, such that the vectors with larger amplitude contribute more to the probability distribution of the vector angle *θ*. The *θ* distributions for the rod- and gravel-NMC, before and after considering the vector amplitude, are shown in Fig. 4d, from which we observe a discernible increment at around 90° for the rod-NMC and negligible change for the gravel-NMC. Figure 4d indicates that the rod-NMC has a larger vector size at around 90°, whereas the gavel-NMC has almost the same vector size at each angle. Figure 4b, d indicate that the rod-NMC has more radially distributed Ni valence state, which is guided by the radially distributed crystallographic orientations of the grains. Figure 4c, d illustrate that the gravel-NMC has relatively random charge distribution as a result of the randomly oriented primary grains. Furthermore, we investigated the vector size distribution as a function of *θ*. We divided the vectors (with *θ* equals 0–90°) into nine equally spaced *θ* groups and plotted the vector size distributions of the respective groups in Fig. 4e for the rod-NMC and in Fig. 4f for the gravel-NMC. The rod-NMC has a slightly smaller vector size comparing with the gravel-NMC, which means the charge heterogeneity is smaller in the rod-NMC. The relative probability of the vectors with larger angles are also much larger for the rod-NMC, while gravel-NMC has very similar vector size distribution at large angles. This size distribution further supports that the crystallographic orientation can affect the charge distribution in polycrystalline layered oxides. We calculated the averaged vector size as a function of *θ* and the result is shown in Fig. 4g. It is evident that the rod-NMC has less charge heterogeneity. The rod-NMC has less stress and a lower degree of charge heterogeneity based on the finite element modeling (FEM, Supplementary Fig. 14, Supplementary Note 1) and 3D TXM, which might help mitigate the polarization and retain capacity.

## Discussion

Polycrystalline nickel-rich layered oxides have recently risen to the top of competitive materials to enable the next-generation, high-energy Li batteries toward 500 Wh kg^−1^. The chemomechanical instability of these materials has remained a key challenge that prohibits their implementation in practical batteries. The chemomechanical breakdown can significantly increase the specific surface area and accelerate the electrode-electrolyte side reactions^26^. These side reactions unambiguously contribute to the surface oxygen loss, electrolyte decomposition, cell impedance buildup, and capacity fading^29^. Therefore, enhancing the chemomechanical properties of polycrystalline cathodes represents a critical research frontier in developing high energy, stable, and safe Li batteries. Grain engineering in polycrystalline materials provides a large playground to modulate the materials properties beyond controlling the chemical composition, and electronic and crystal structures. In particular, the anisotropic ion conducting pathways in layered oxides make the grain crystallographic orientation a critical factor in determining the modality of the redox reactions in these materials. To date, there have been several studies highlighting the importance of controlling the grain crystallographic orientation to improve the cycling stability of Li and Na layered cathode materials^4,5,34–37^. However, the underlying mechanism how the charge distribution changes as a function of the grain orientation has not been studied. Such a study would have offered valuable insights into designing microstructure-controlled battery particles for improving battery cycle life. As reported in recent studies, the charge heterogeneity prevails in polycrystalline electrode particles even after an extend period of relaxation^18,46^. The studies have concluded that the grain boundaries may play a role in directing the Li ion conducting pathways. The present study provides an unprecedented mechanistic understanding of how the charge distribution is guided by the grain crystallographic orientation in polycrystalline battery materials through a suite of advanced synchrotron spectroscopic, diffraction, and imaging techniques. We systematically investigated the charge distribution in two Li layered oxides that had the similar chemical composition, global crystal structure, and surface and bulk electronic structures but different crystallographic orientations. These cathodes exhibited comparable initial discharge capacity but noticeable differences in cell polarization and cycle life. Overall, the radially aligned grains enabled relatively more stable battery performance, which is consistent with recent studies^5,6^. The electronic characterization, through soft and hard XAS, revealed that these two materials showed nearly identical global state-of-charge. However, the spatially resolved TXM and the associated mathematical quantification demonstrated that the radially align grains created a charge distribution that resembled the grain orientation and more importantly led to relatively smaller charge heterogeneity. Moreover, our finite element modeling (FEM) simulation demonstrates that the delithiation process generates less stress between the grains in the rod-NMC, possibly due to the cooperative alignment of the grains (Supplementary Fig. 14, Supplementary Note 1). This work provides a strong mechanistic incentive to improve the stability of polycrystalline battery materials through proper engineering of grain morphology and orientation.

## Methods

### Materials

The precursor for the rod-NMC was obtained from Shuangdeng Group Co, Ltd. The XRD and SEM images of the precursor are provided in Supplementary Fig. 15. Subsequently, the precursor was calcined at Virginia Tech using the following protocol: the precursor was dried in a vacuum oven at 120 °C for 12 h before the calcination. The dried precursor was mixed with LiOH (5% extra was added to compensate for Li loss) thoroughly and calcined under pure oxygen flow at 2.0 L min^−1^. The sample was heated at 5 °C min^−1^ to 160 °C and held there for 1 h. The sample was then heated at 5 °C min^−1^ to 460 °C and held there for 2 h. Then the sample was heated at 5 °C min^−1^ to 750 °C and remained there for 6 h. Finally, the furnace was cooled at 5 °C min^−1^ to 25 °C still under constant oxygen flow to obtain the final powder. The gravel-NMC was provided by the U.S. Department of Energy’s (DOE) CAMP (Cell Analysis, Modeling and Prototyping) Facility, Argonne National Laboratory.

### Electrochemical performance

Composite electrode were prepared using 90% active material, 5% polyvinylidene fluoride, and 5% acetylene carbon black in N-methyl-2-pyrrolidone and then cast onto carbon-coated aluminum foil current collectors. The electrodes were dried under vacuum at 120 °C. The electrodes have a loading of ~4.5 mg cm^−2^. CR2032-type coin cells were assembled in an argon-filled glove box using the electrode as the cathode and Li metal as the anode. The cathode and anode were separated with a glass fiber separator which was soaked with an electrolyte of 1 M LiPF_6_ dissolved in a 3:7 weight ratio of ethylene carbonate/ethyl methyl carbonate with 2 wt% vinylene carbonate. All the electrochemical testing was performed using a Neware battery testing system. 1C was defined as fully charging the cathode in 1 h, with a specific capacity of 200 mAh g^−1^.

### Material characterization

The energy-dispersive X-ray spectroscopy (EDS) were conducted on an FEI Quanta 600 FEG at an accelerating voltage of 15 kV. The inductively coupled plasma-mass spectrometry (ICP-MS) was performed on a SPECTRO ARCOS ICP-AES analyzer. The morphologies of the materials were investigated using LEO (Zeiss) 1550 field-emission scanning electron microscopy (SEM) at an accelerating voltage of 5 kV. To expose the interior morphologies of the cathode particles, polishing or weak acid etching were used. For the polishing method, cathode particles were dispersed in pre-mixed epoxy (Loctite). The interior of the secondary particles were exposed using sand papers after the epoxy was dried for 48 h. For the weak acid etching method, 100 mg cathode particles were dispersed in 50 mL boric acid (pH = 4.0) solution with gentle agitation. The cathode mixture was centrifuged and dried in a vacuum oven overnight. The cross-sectional samples prepared for transmission electron microscopy (TEM) characterizations were conducted on an FEI Helios NanoLab 600i focused ion beam (FIB) operated at 2–30 kV. To protect the sample from beam damage, 1.5-μm-thick Pt layer was deposited on a particle surface to void the Ga ion-beam damage in the subsequent lift-out and thinning process. The specimen was thinned to <200 nm. The SEM images were collected in the process of sample thinning. STEM-HAADF imaging and nano-beam diffraction (NBD) were performed on a 300 kV Titan G2 60-300 microscope equipped with a probe spherical aberration corrector. X-ray diffraction (XRD) was performed at beamline 11-3 of Stanford Synchrotron Radiation Lightsource (SSRL). Transmission XRD ring patterns were detected. LaB_6_ patterns were collected as reference data for calibration, and exposure time was only 0.5 s for the samples to avoid any saturation. The synchrotron XRD results (wavelength = 0.9762 Å) are transferred to Cu K-α based lab-XRD results (wavelength = 1.5406 Å) as shown in Fig. 1g. XRD patterns on the precursor and pristine powder materials were acquired through a Rigaku MiniFlex II diffractometer with a Cu Kα radiation (*λ* = 1.54 Å) X-ray source. Data acquisition was performed from an initial 2*θ* of 10° to the final 2*θ* of 90° with a scan rate of 0.20° per minute. The crystal structure refinement was carried out through the Rietveld method, as implemented in the Fullprof software package. Hard X-ray absorption microscopy (XAS) measurements were performed on the electrodes in transmission mode at the beamline 20-BM-B of the advanced photon source (APS) at Argonne National Laboratory. The incident beam was monochromatized by using a Si (111) fixed-exit and a double-crystal monochromator. Energy calibration of each spectrum was made by aligning the first derivative maximum of a reference Ni hard XAS spectra collected simultaneously from the metal foils in the reference channel. The edge energy is the absorption energy at which the absorbance is 0.5. Depth-resolved confocal hard XAS, with an illumination area around 36 μm^2^, was performed at the beamline 20-BM-B of the APS at Argonne National Laboratory. The thin film electrode sample was moved in one direction every 5 μm to get depth-resolved XAS. Soft XAS measurements were performed on the 31-pole wiggler beamline 10-1 at SSRL using a ring current of 350 mA and a 1000 L mm^−1^ spherical grating monochromator with 20 μm entrance and exit slits, providing ∼10^11^ ph s^−1^ at 0.2 eV resolution in a 1 mm^2^ beam spot. Data were acquired under ultrahigh vacuum (10^−9^ Torr) in a single load at room temperature using total electron yield (TEY), where the sample drain current was collected, and in the fluorescence yield (FY), where a silicon diode (IRD AXUV100) was used to collect the FY positioned near the sample surface. All spectra were normalized by the current from freshly evaporated gold on a fine grid positioned upstream of the main chamber. After the cell was charged to the designated state of charge, the cells were transferred to an Ar-filled glove box (water level < 0.5 ppm, oxygen level < 0.5 ppm) immediately. The cathode disk was collected, rinsed with dimethyl carbonate, dried, and then sealed in Mylar aluminum bags in the glove box. The cathode particles were sealed into the TXM capillary carefully using a cotton covered wooden stick. The transmission X-ray microscopy (TXM) was performed at beamline 6-2c at SSRL. An in-house developed software package known as TXM-Wizard was used for the analysis of all the tomography results^47,48^. To be specific, the XANES spectrum of each pixel per voxel is generated by imposing and aligning the absorption results at different energy. After the normalization procedure (Supplementary Fig. 16), the pre-edge of the spectra is set to be zero and the post-edge is set to be one. To quantify the relative energy shift of the spectra, the edge energy, which is defined as the energy level that corresponds to normalized intensity of 0.5, is extracted to benchmark the relative valence state of Ni (a proxy for the local state of charge). Subsequently, the edge energy is used to generate the valence state maps. The 2D nanodomain valence gradient vectors were calculated by adding two partial valence gradient vectors (e.g., **u**_***x***_ and **u**_***y***_ in XY plane). The vector direction presents the local distribution of Ni valence states and the vector magnitude is proportional to local valence states gradient. The definition of 3D nanodomain valence gradient vectors are discussed in the Results and Discussion sections.

### Finite element modeling

To simulate Li diffusion and stress evolution in the NMC particles, a more tractable 2D plane strain model was built. The NMC particle was represented by a circular domain composed of multiple grains. The *a*-axis of each grain in the rod-NMC was radially aligned while the grains in gravel-NMC were randomly packed. The Li diffusion and associated strain were highly anisotropic. Li diffusivities along the *a*-axis and *c*-axis were set as Da = 7 × 10^−15^ m s^−2^ and Dc = Da/10 = 7 × 10^−16^ m s^−2^, respectively. The diffusion induced strains at 4.5 V in the *a*-axis (and *b*-axis, out-of-plane) and *c*-axis are set as −2.1% and −3.7%, respectively, according to a prior study of the Bragg peak shifting in in-situ XRD scanning experiments^25^. A zero-displacement condition at the center of NMC particle was used to prevent the rigid motion. A Li outflux calculated based on the cyclic rate of C/5 was imposed on the surface of the NMC particle. The governing equations for the kinematics of deformation and the kinetics of diffusion were solved using the commercial software COMSOL Multiphysics V5.3. Details of the numerical framework can be found in a previous work^50^.

## Supplementary information

Supplementary Information

## Data Availability

The data that support the findings of this study are available from the corresponding author upon reasonable request.

## Code availability

The code that support the findings of this study are available from the corresponding author upon reasonable request.
