# Supplementary material for: Charge distribution guided by grain crystallographic orientations in polycrystalline battery materials
Source: Nat Commun. 2020 Jan 8;11:83. doi: 10.1038/s41467-019-13884-x (PMC6949258; doi:10.1038/s41467-019-13884-x)
Supplement: Supplementary file 1 — Supplementary Information [file 41467_2019_13884_MOESM1_ESM.pdf]

Charge Distribution Guided by Grain Crystallographic Orientations in Polycrystalline Battery Materials

Xu et al.

Supplementary Figures

a

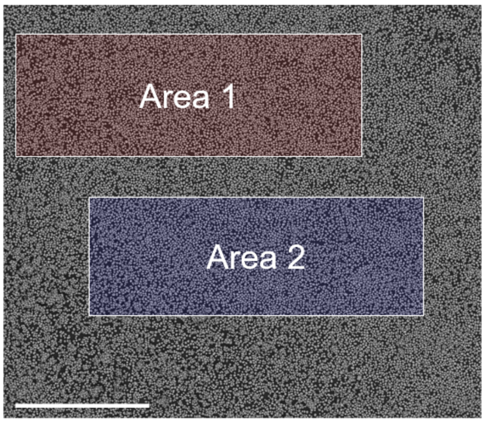

b

Sample area	Atomic Ratio (%)		
	Ni	Co	Mn
Area 1	82.61	12.19	5.20
Area 2	82.35	12.37	5.28

e

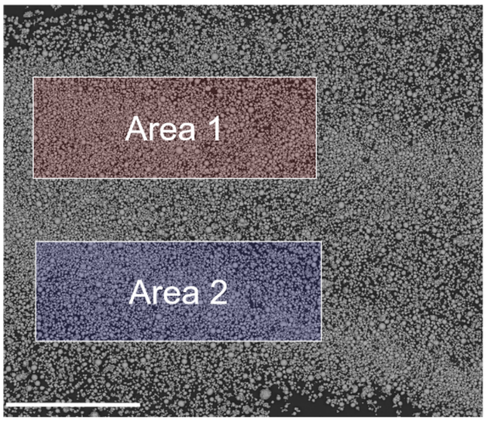

f

Sample area	Atomic Ratio (%)		
	Ni	Co	Mn
Area 1	80.04	10.56	9.41
Area 2	80.45	10.52	9.03

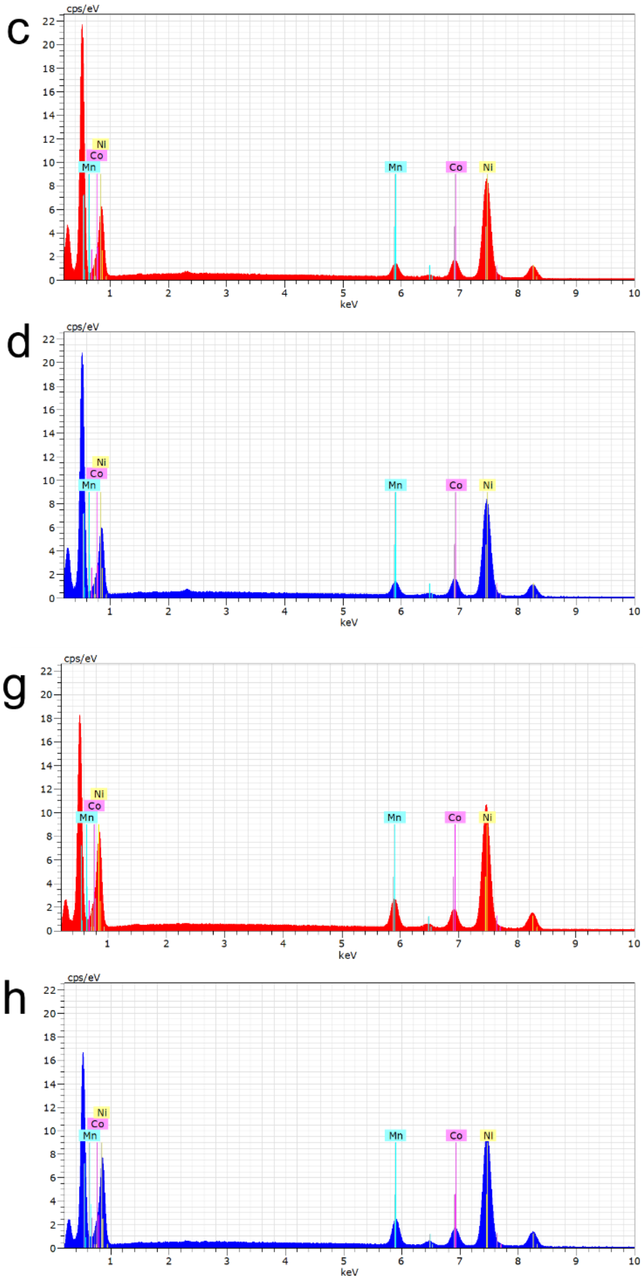

Supplementary Figure 1 | EDS result of the two NMCs. Low magnification SEM images of the (a) rod-NMC and (e) gravel-NMC, where two large areas were investigated (numbered as 1 and 2), respectively. EDS results of the (c) area 1 and (d) area 2 in (a). The transition metal atomic ratios in (a) are summarized in (b). EDS results of the (g) area 1 and (h) area 2 in (e). The transition metal atomic ratios in (e) are summarized in (f). Both scale bars in (a) and (e) are 500 μm.

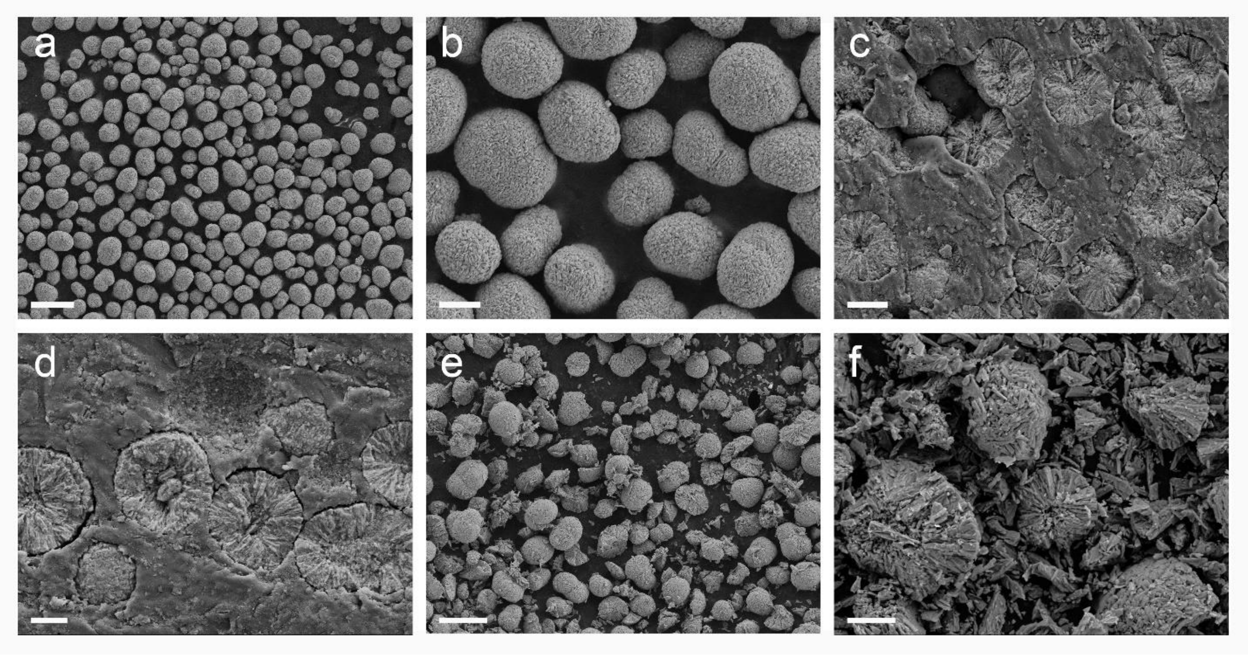

Supplementary Figure 2丨 **Characterization of the rod-NMC morphologies.** (a-b) Overall morphology of the rod-NMC. (c-d) Interior SEM images of rod-NMC using the polishing method as described in the “Method” part. (e-f) Interior SEM images of rod-NMC via the weak acid etching method as described in the “Method” part. The scale bars in a-f are 20 μm, 5 μm, 5 μm, 3 μm, 15 μm, and 3 μm, respectively.

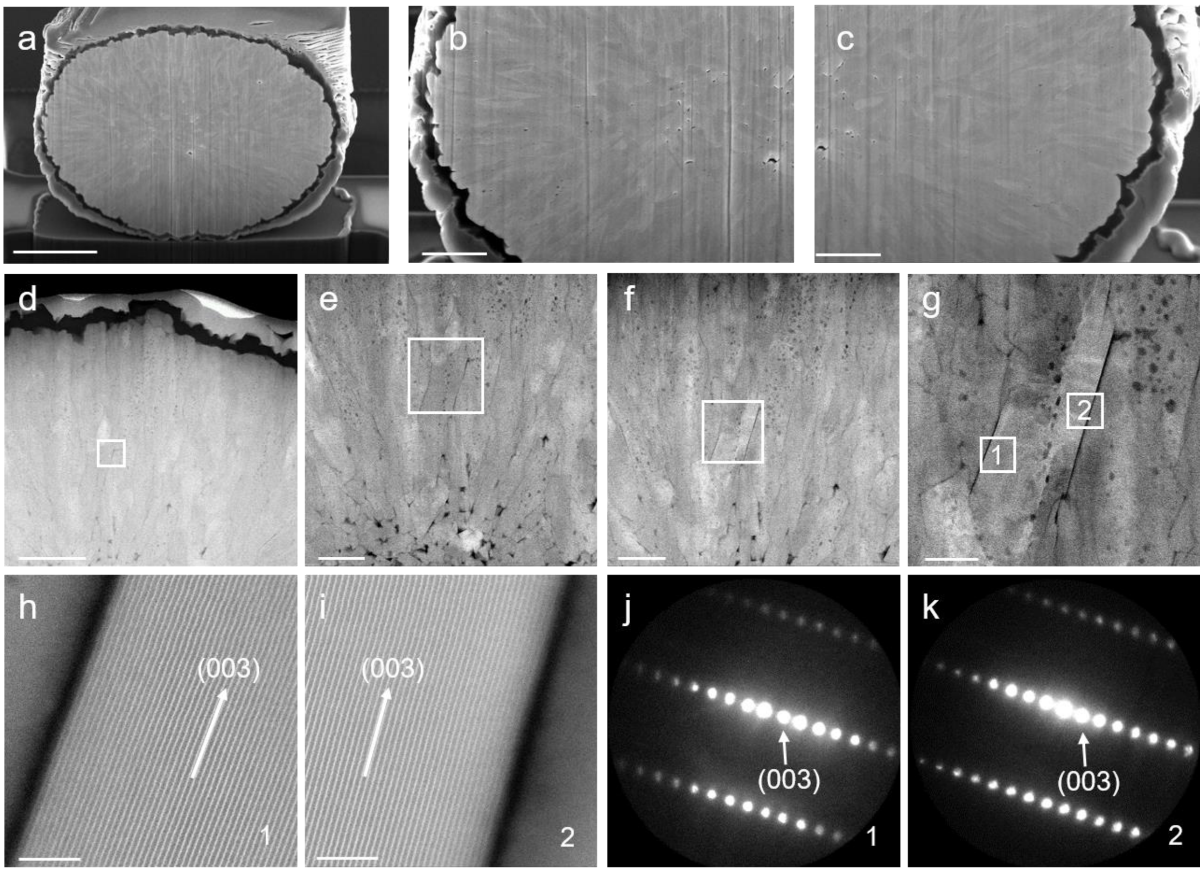

Supplementary Figure 3 | Investigation of the rod-NMC crystallographic texture using FIB-SEM, STEM-HAADF, and NBD. (a-c) Cross section SEM images of the rod-NMC sample, which shows the rod-shaped grains are along the radial direction. (d-g) TEM images of the cross section, where two zones (named as “1” and “2” in (g)) were selected for the subsequent study. (h-i) STEM-HAADF images of zone 1 and zone 2 in (g), respectively. The (003) fringes in h-i are along the radial direction, which are proved by the NBD in (j) and (k), respectively. The STEM-HAADF images and NBD patterns of the rod-NMC are shown in relation to the secondary particle, which confirm that the Li channels are along the radial direction. The scale bars in a-i are 3 μm, 1 μm, 1 μm, 500 nm, 500 nm, 500 nm, 200 nm, 5 nm, and 5 nm, respectively.

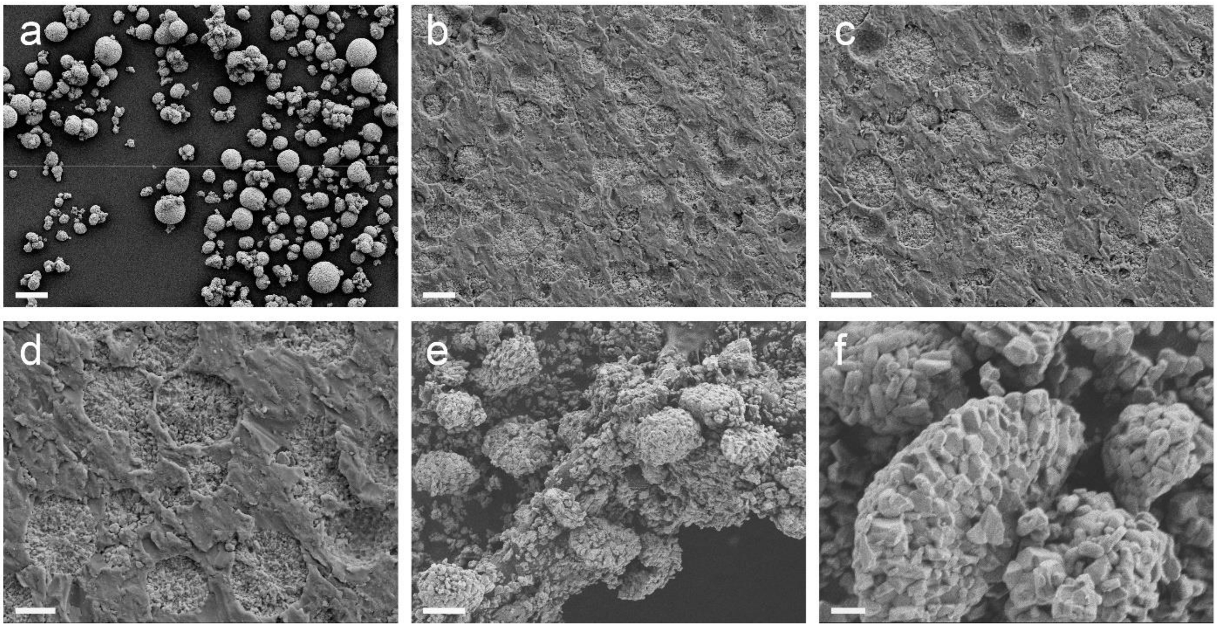

Supplementary Figure 4 | Characterization of the gravel-NMC morphologies. (a) Overall morphology of the gravel-NMC. (b-d) Interior SEM images of the gravel-NMC using the polishing method as described in the “Method” part. (e-f) Interior SEM images of the gravel-NMC by the weak acid etching method as described in the “Method” part. The scale bars in a-f are 20 μm, 10 μm, 10 μm, 5 μm, 5 μm, and 1 μm, respectively.

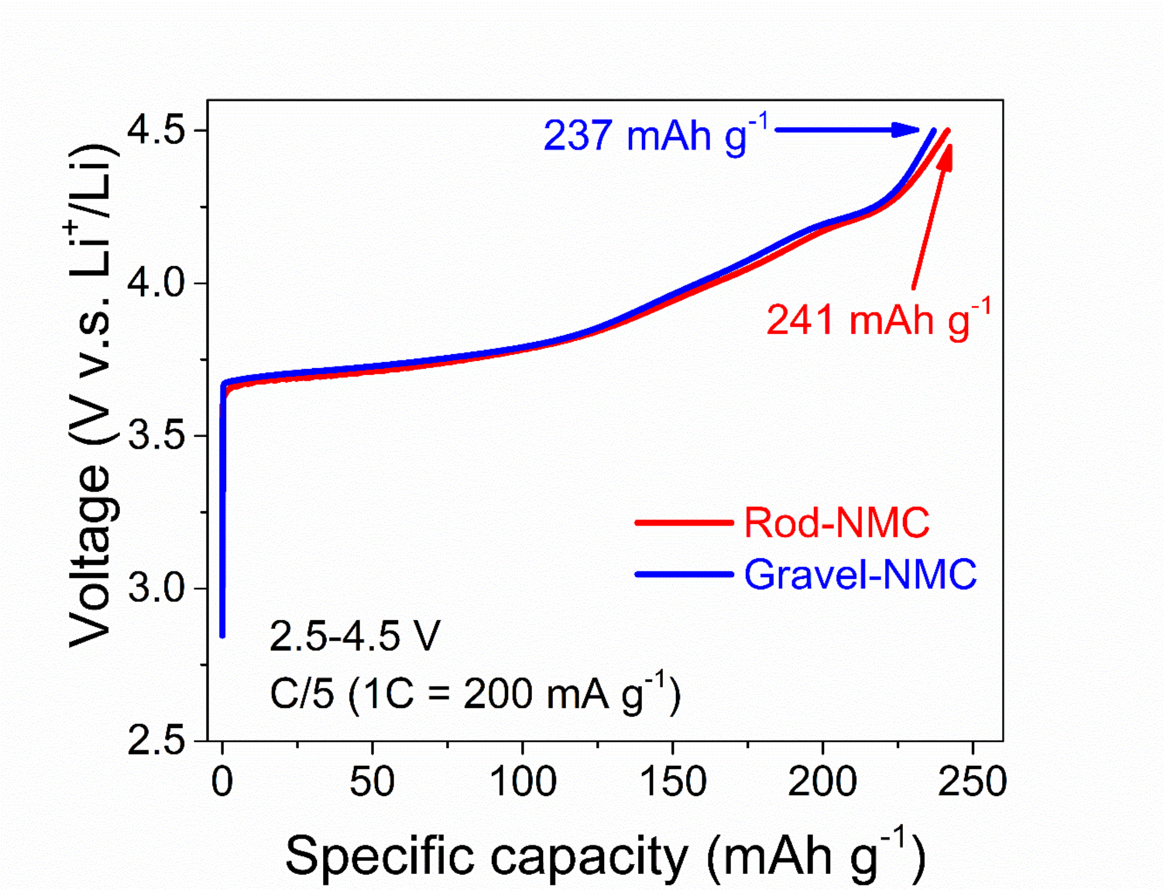

Supplementary Figure 5 | First charge profile of the rod-NMC and gravel-NMC at 2.5-4.5 V at C/5 (1C = 200 mA g^{-1}). The rod-NMC and gravel-NMC have similar charge capacity in the first charge. The first charged rod-NMC and gravel-NMC were used for the XRD, XAS and TXM experiments as described in the "method" part.

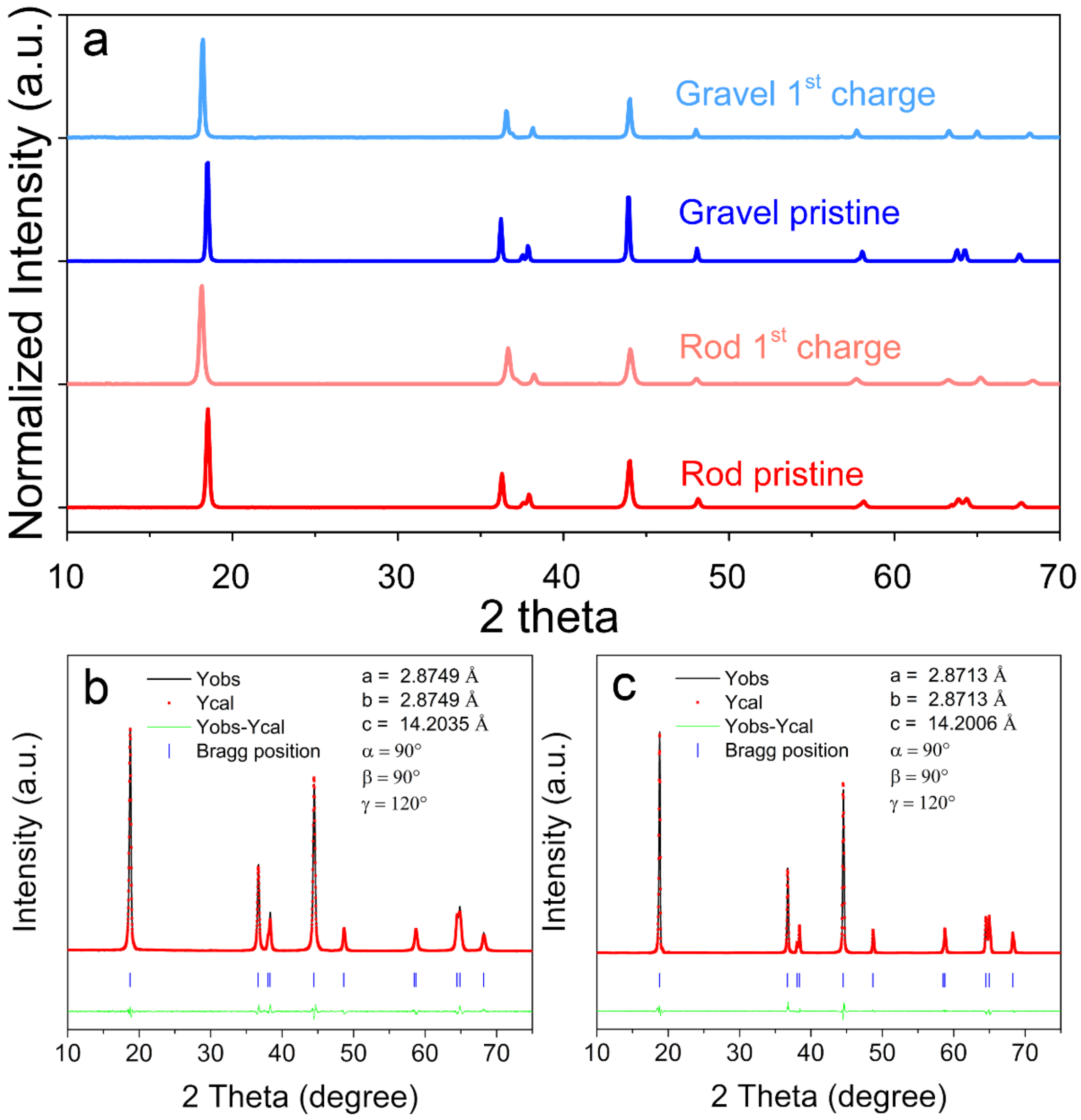

Supplementary Figure 6 │Full XRD patterns of the rod- and gravel-NMCs. (a) Synchrotron XRD patterns of the rod- and gravel-NMCs in the pristine and first charged state, based on the electrode samples sealed in Kapton tapes. The 2 theta is based on the transformation from synchrotron wavelength to lab based Cu K-α wavelength as described in the "method" section. XRD Rietveld refinement of the (b) pristine rod-NMC and (c) pristine gravel-NMC based on the lab powder XRD. The lattice parameters from the Rietveld refinement are included as insets in b-c.

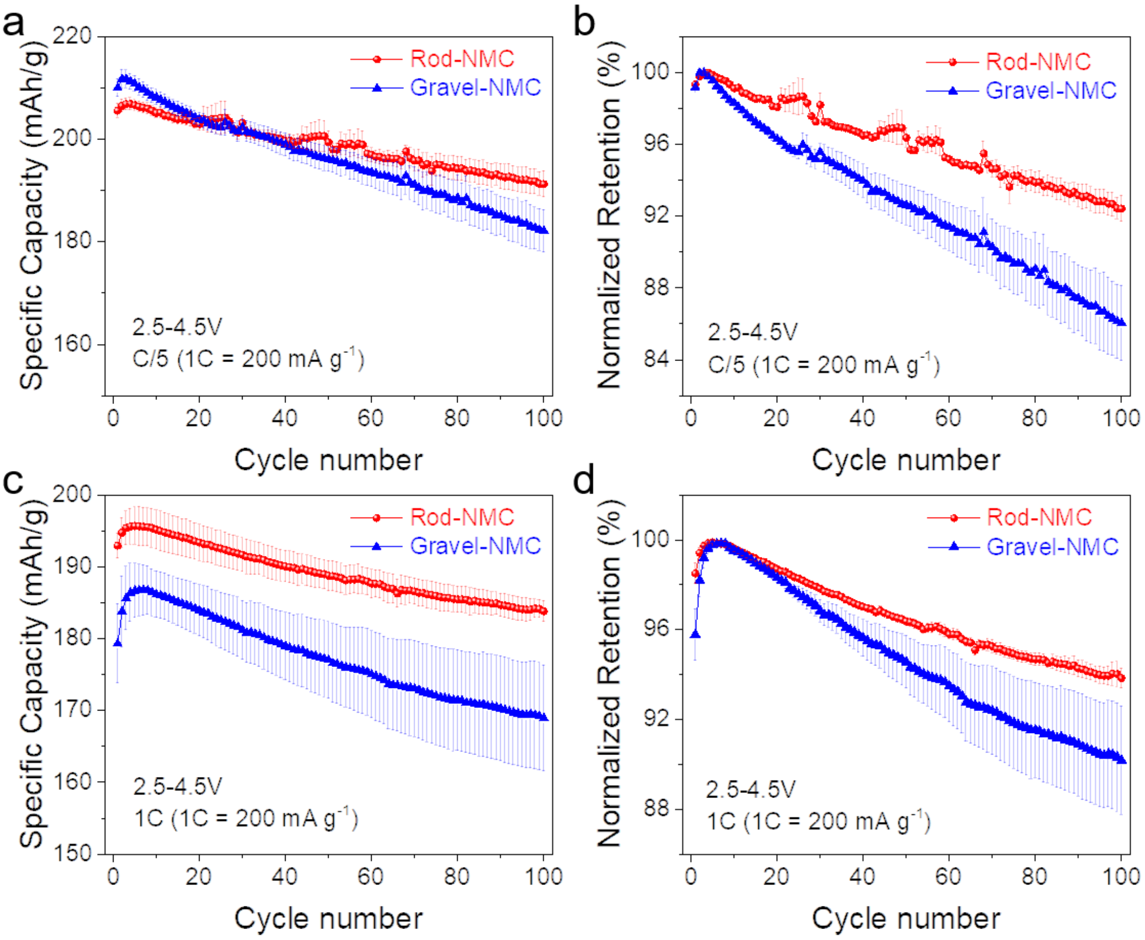

Supplementary Figure 7 | Cycling performance comparison of the rod-NMC and gravel-NMC at C/5 and 1C (1C = 200 mA g^{-1}). The (a) specific discharge capacity and (b) normalized capacity retention of the rod- and gravel-NMCs at C/5. The (c) specific discharge capacity and (d) normalized capacity retention of the rod- and gravel-NMCs at 1C. The capacity retention is calculated using the maximum capacity as the 100% reference. The performance measurements are based on three coin cells and error bars are included.

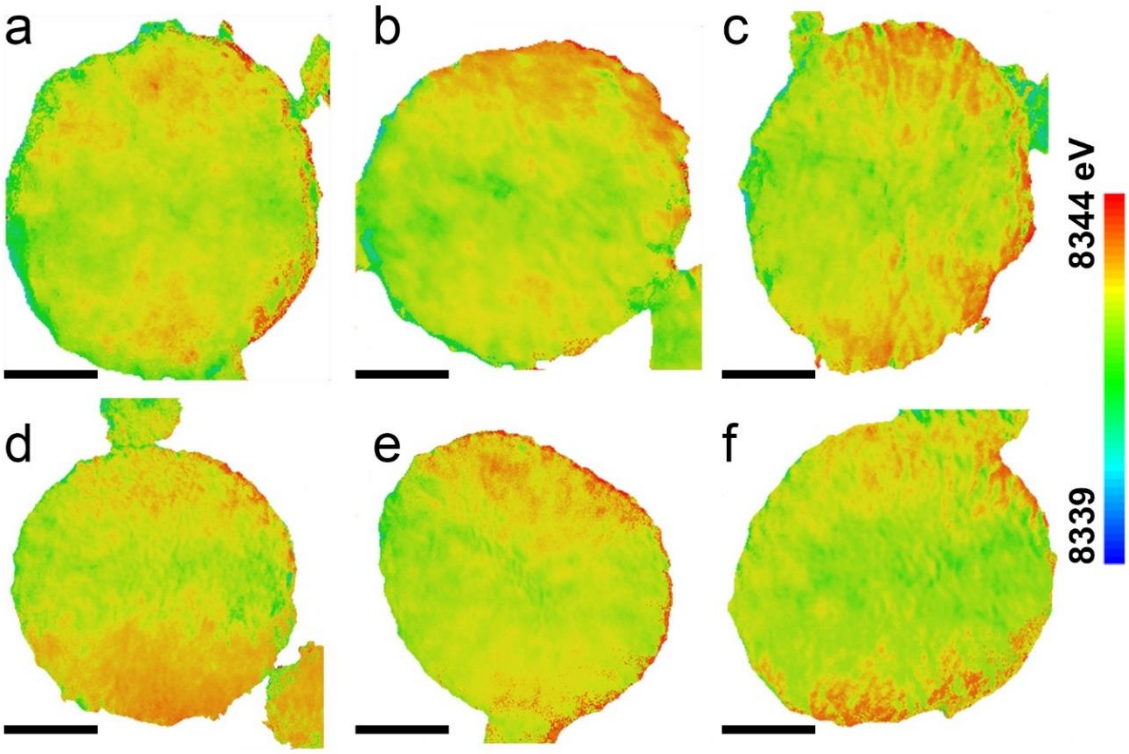

Supplementary Figure 8│ 2D TXM of the NMC sample in the first charged state (cut-off voltage is 4.5 V) at a charging rate of C/10. a-f are six independent particles. The local Ni valence states are color coded, in which red represents more oxidized Ni nanodomains and blue stands for less oxidized Ni nanodomains. The scale bars in a-f are all 3 μm.

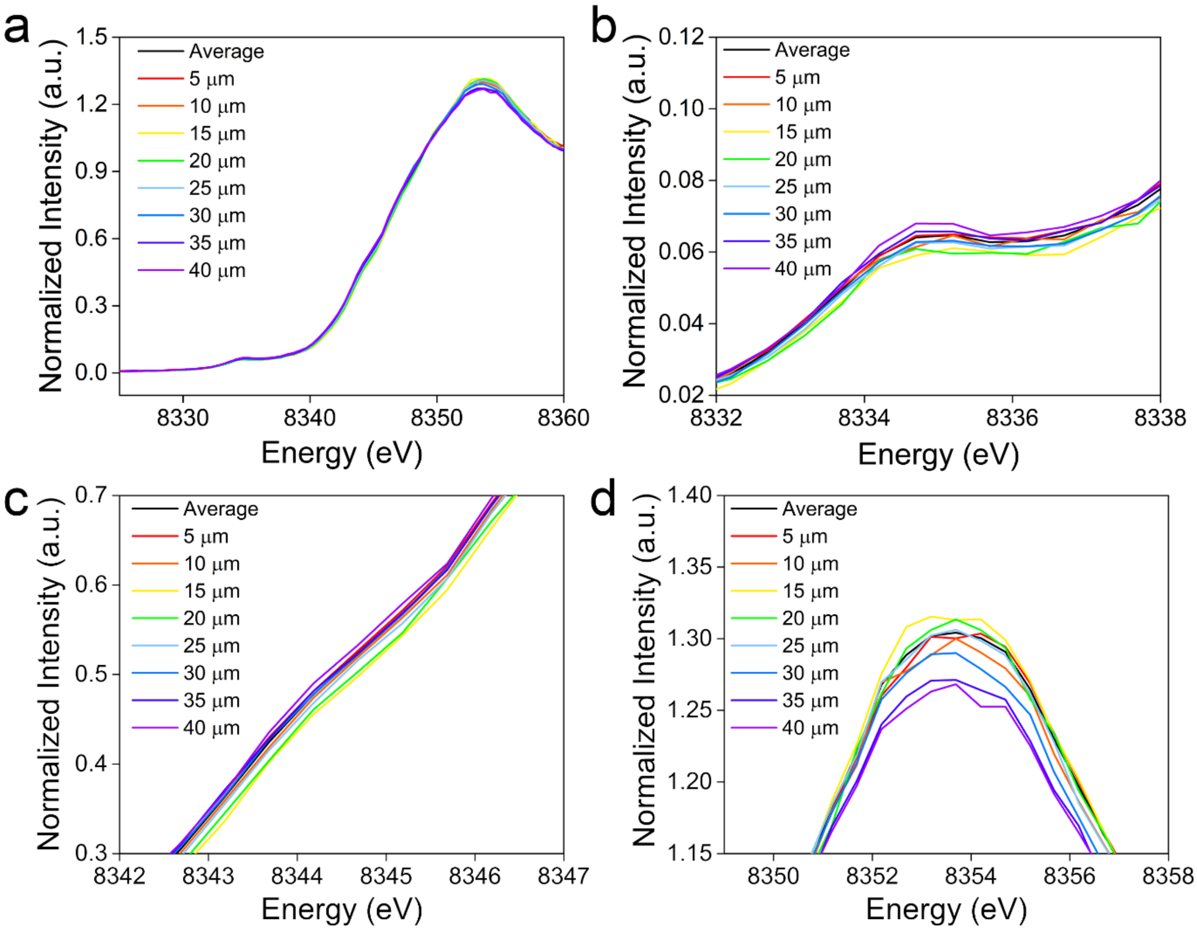

Supplementary Figure 9 | Depth resolved confocal hard XAS in the first charged state. The cathode thin film has a thickness around 40 μm. The depth in a-d is the distance from the probed region and the electrode surface. The average line is the overall hard XAS for the whole electrode. (a) Overall depth sensitive confocal hard XAS result. (b) Pre-edge, (c) edge, and (d) whiteline of the depth resolved confocal hard XAS results in (a).

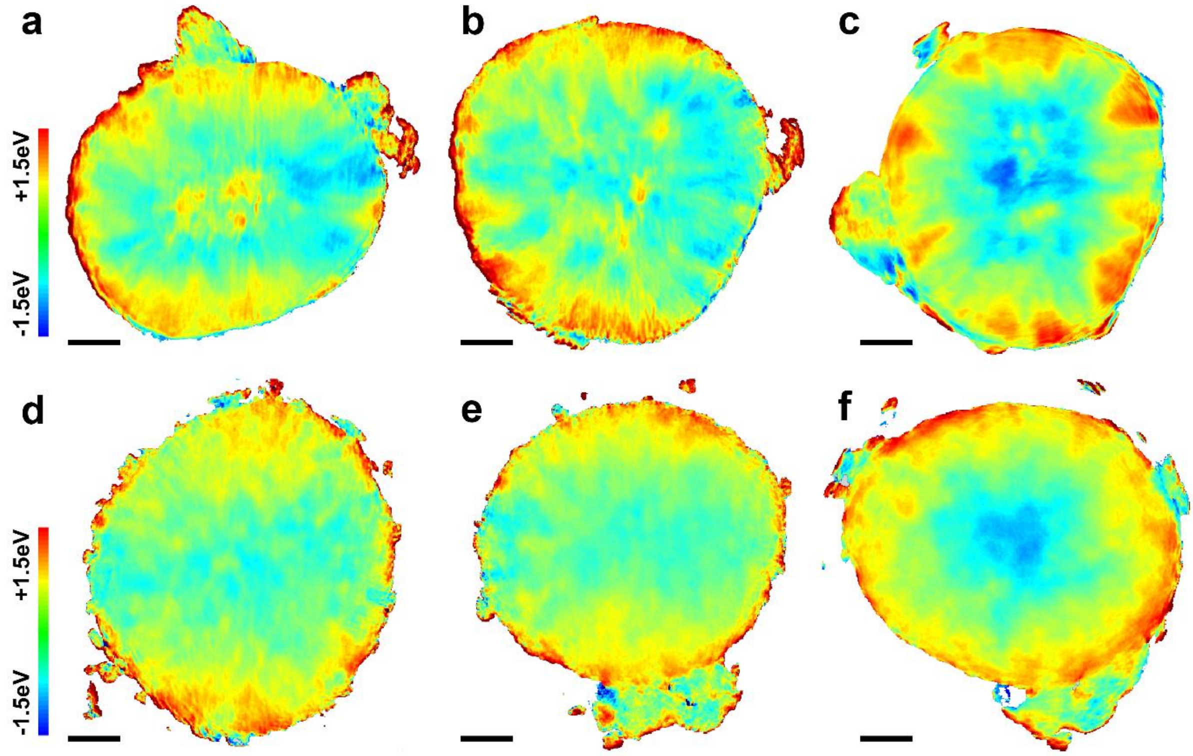

Supplementary Figure 10 | 2D TXM results of the rod- and gravel-NMCs in the first charged state. Projection of the 3D TXM results of rod-NMC in (a) XY, (b) YZ, and (c) XZ direction. Projection of the 3D TXM results of gravel-NMC in (d) XY, (e) YZ, and (f) XZ direction. All of the scale bars in a-f are 2 μm. The Ni K-edge energy are color coded, in which blue and red stands for lower edge energy and higher edge energy, respectively.

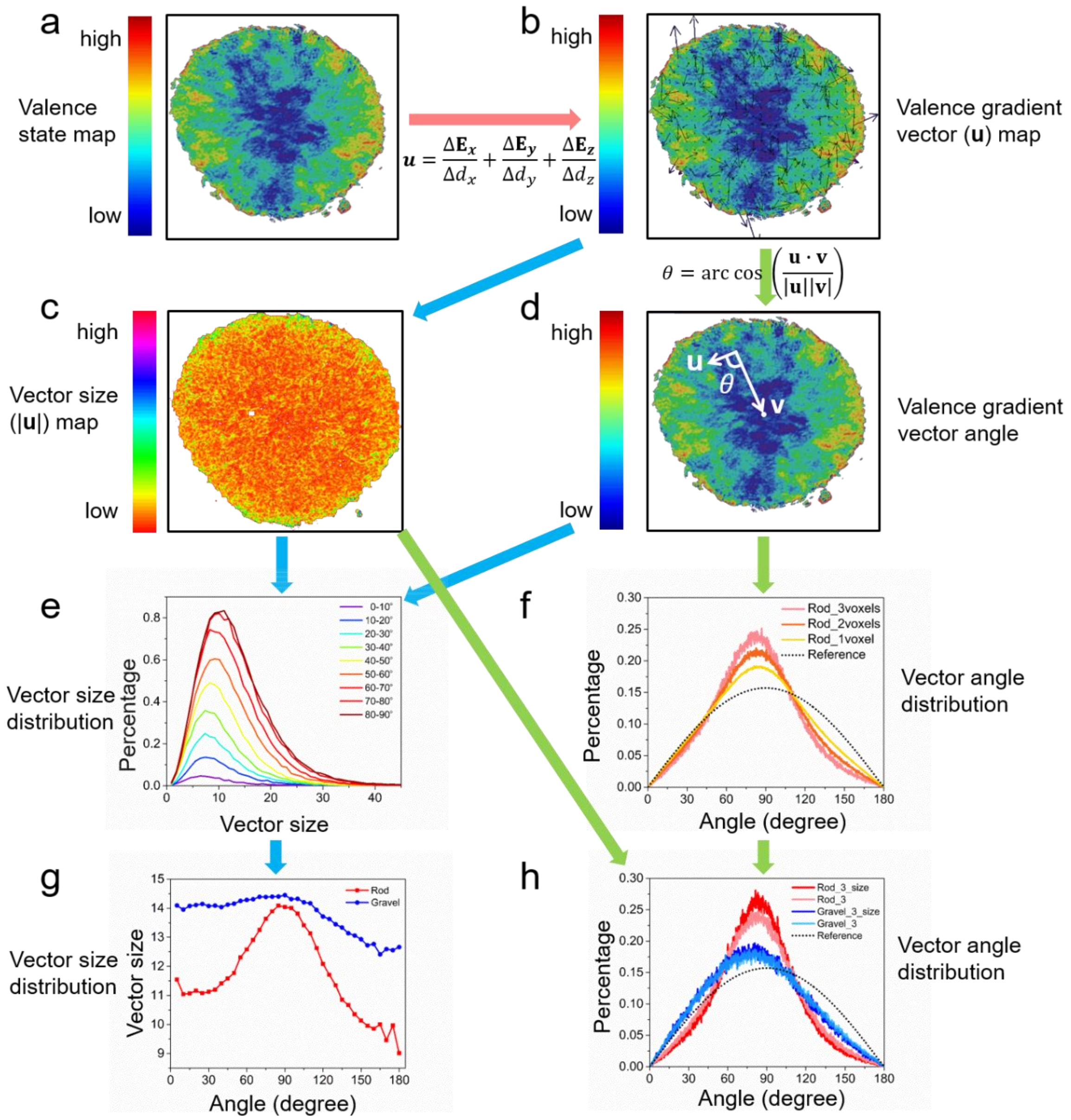

Supplementary Figure 11 | Explanation of TXM data analysis process. (a) Valence state map in Fig. 3a and Fig. 3d. (b) Valence gradient vector calculation based on the model in Fig. 4a. (c) Extraction of valence gradient vector size. (d) Valence gradient vector angle calculation based on the model in Fig. 4a. (e) Vector size distribution calculation based on (c) and (d). (f) Vector angle distribution (before considering vector size) based on (d). (g) Averaged vector size distribution based on (e). (h) Vector angle distribution (after considering vector size) based on (c) and (f).

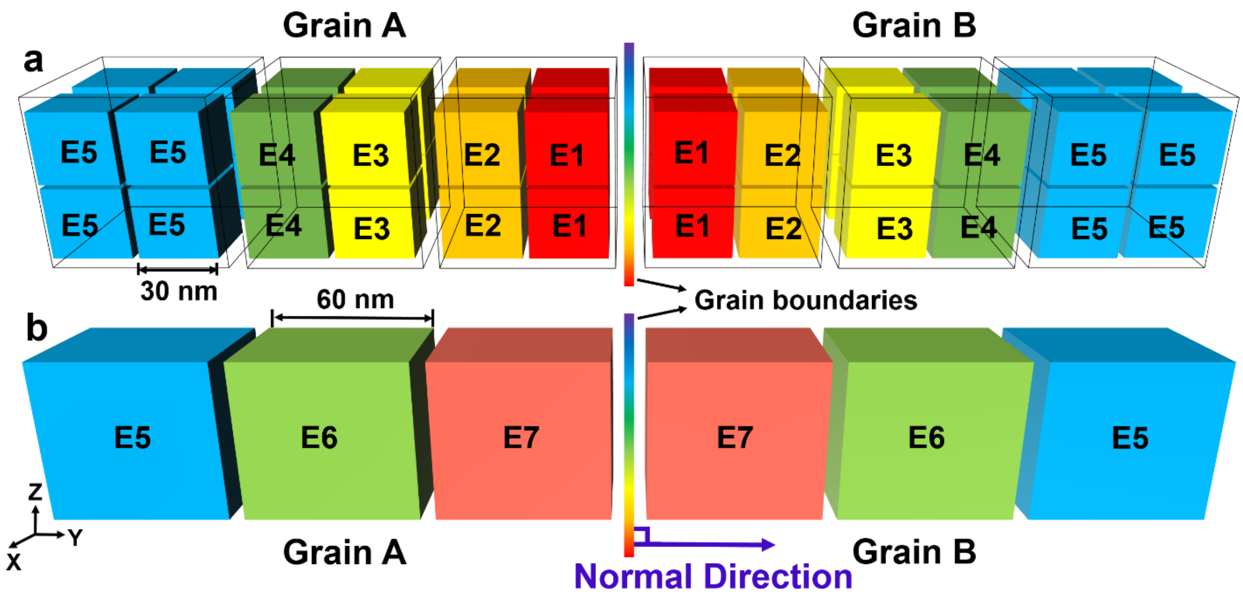

Supplementary Figure 12 | Amplifying grain boundary contribution by averaging voxels. Valence gradient model in the case of (a) 1 voxel (30*30*30 nm^3) or (b) 8 voxels (60*60*60 nm^3, merging 2 voxels in the x, y and z directions) as a single unit on two neighboring grains (Grain A and Grain B). The Ni K-edge absorption energies of the voxels are color-coded and denoted as E1 to E7. In (a), the voxels near the grain surface are higher in valence states while moving inward to a grain the valence values decrease (E1>E2>E3>E4>E5). Averaging does not change the valence gradient along the grain boundary surface because the valence states of the voxels along grain boundaries are similar (represented as E1 in (a)). Likewise, averaging does not change the valence gradient in the grain bulk due to the similar valence states (represented as E5 in (a)). However, averaging voxels along the normal direction to the boundary surfaces increase the valence gradient near the grain boundaries (represented as E1 to E4 in (a)). Therefore, the theta calculated after averaging voxels will be more along the normal directions, which leads to sharper peaks in Fig. 4b. In conclusion, merging the voxels (i.e., artificially reducing the spatial resolution) can help us to amplify the contribution from regions in the vicinity of grain boundaries.

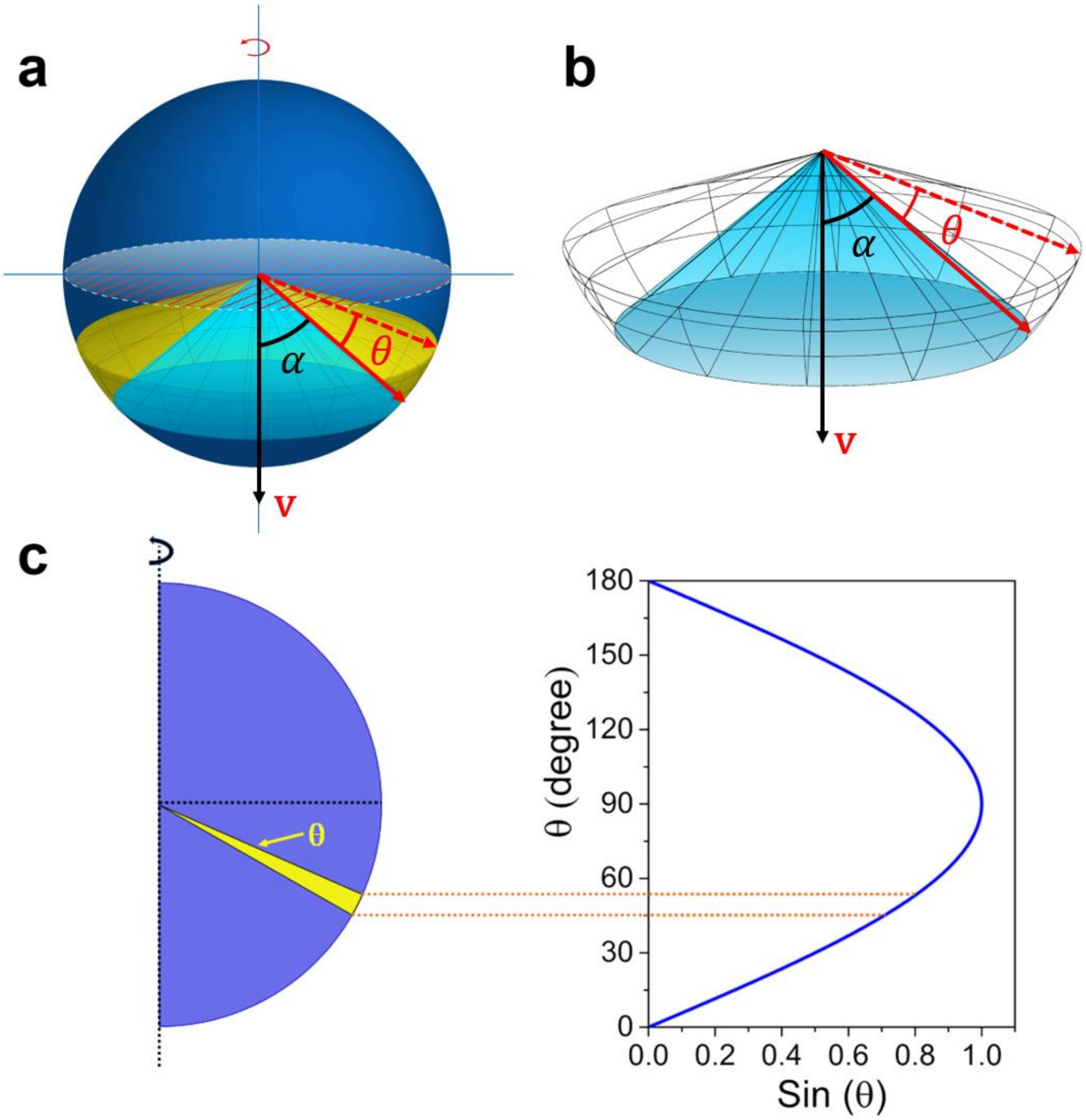

Supplementary Figure 13 | Explanation of the reference data in Fig. 4. (a) The vectors with vector angle between α and $\alpha + \theta$ are in the yellow zone, which is from rotating the sector for 360°. The vector **v** has the same meaning to the one in Fig. 4a. (b) Further explanation of the rotation of the sector in (a). (c) The number of vectors with different θ are proportional to $\sin\theta$. The density of vectors is constant, the number of vectors with different θ is therefore proportional to the volume that these vectors occupy (yellow zone in (a)), which is proportional to $\sin\theta$. The θ value distribution in (c) stands for the arbitrary case when the distribution of the Ni K-edge energy is totally random in a secondary particle.

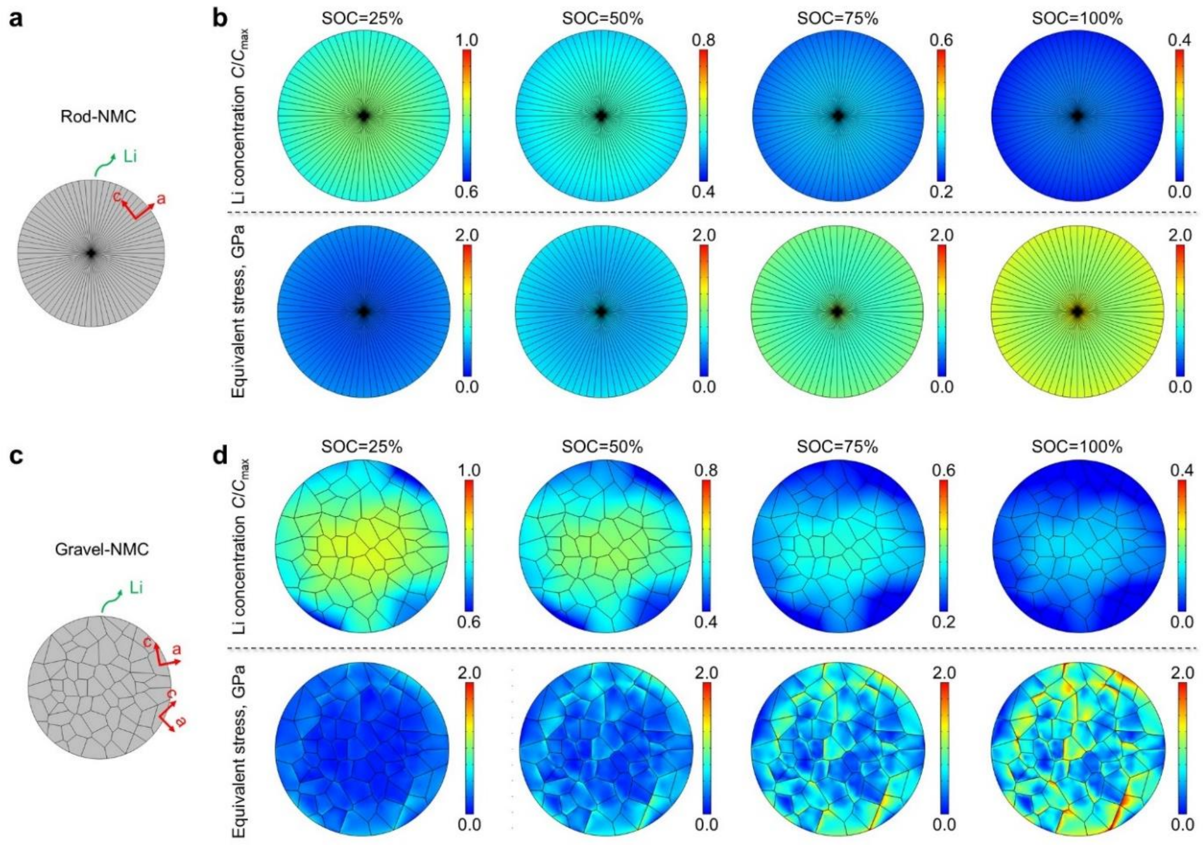

Supplementary Figure 14 | Numerical analysis of Li diffusion and stress evolution in the rod- and gravel-NMCs. (a, c) Finite element models of (a) the rod-NMC consisting of radially aligned grains and (c) gravel-NMC consisting of randomly packed grains. (b, d) Distributions of Li concentration and equivalent stress in the (b) rod- and (d) gravel-NMCs at different SOCs. With the radially aligned grains, the distribution of Li concentration and equivalent stress evolves homogeneously in the rod-NMC during the delithiation. In the gravel-NMC, due to the anisotropic Li diffusion and large mismatch strain, the Li concentration distributes heterogeneously in the particle, and the stress highly concentrates at grain boundaries.

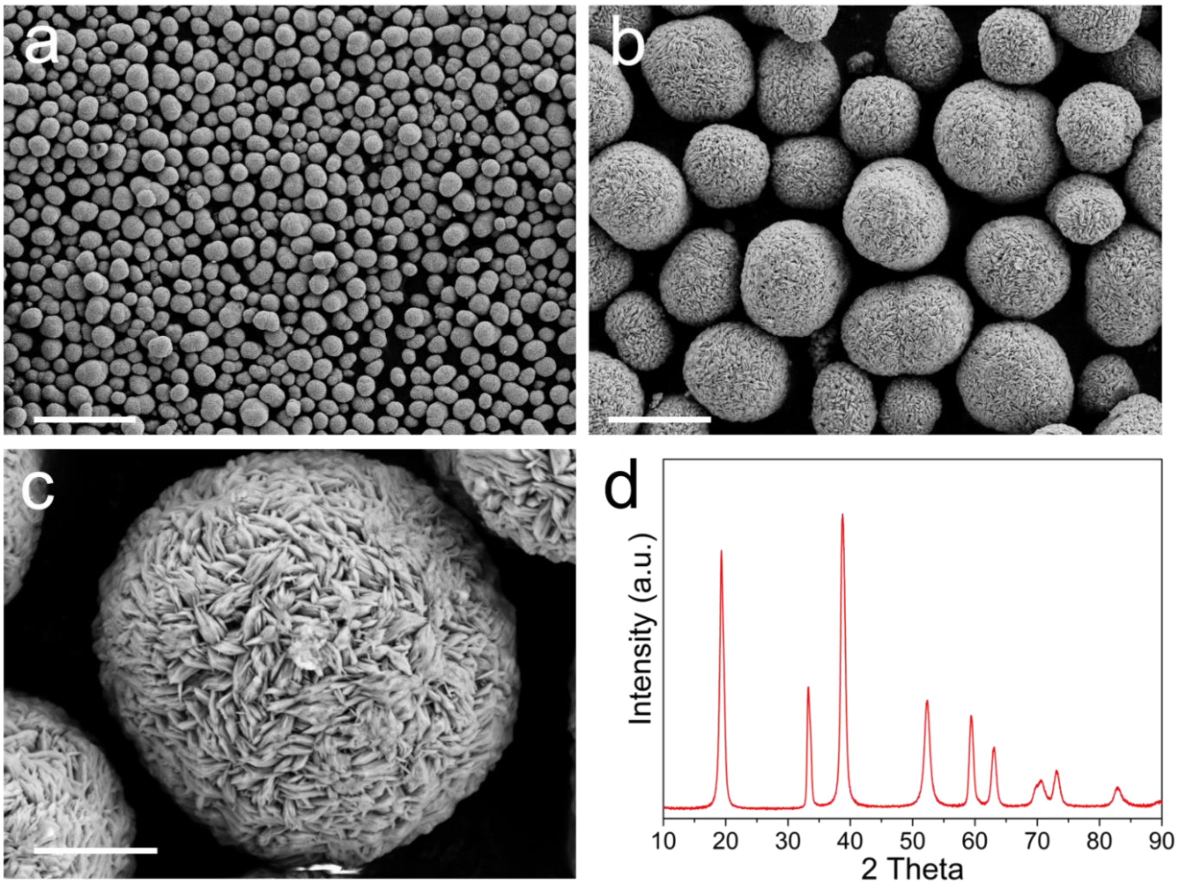

Supplementary Figure 15 | Characterization of the rod-NMC precursor. (a-c) SEM images of the rod-NMC precursor under different magnifications. (d) Powder XRD of the rod-NMC precursor. The scale bars in a-c are 50, 10, and 3 μm, respectively.

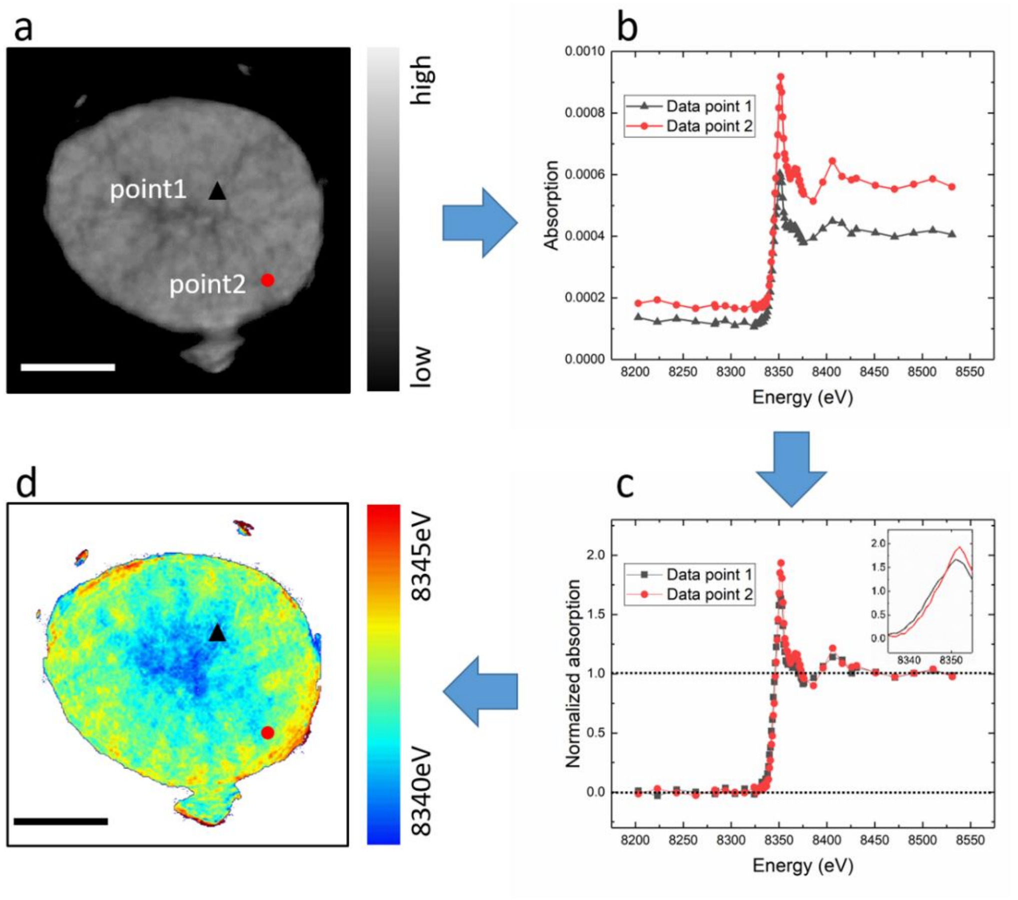

Supplementary Figure 16 | Explanation of the TXM data normalization protocol. (a) An absorption image of an NMC particle at a specific energy, where the intensity represents the absorption and the scale is indicated by the gradient bar to the right of the image. Two domains with different porosity (point 1 with higher porosity while point 2 has lower porosity) were selected to explain the normalization protocol. (b) XANES spectra of the two selected domains before normalization process. The two spectra extracted from (a) could be significantly different in their respective edge jump, indicating difference in the absolute amount of Ni within these two domains (due to sub-pixel level micro-pores and/or different local concentrations of Ni). (c) XANES spectra of the two selected domains after the normalization procedure, which is applied to normalize the absolute intensity and to highlight their chemically relevant spectroscopic fingerprints. (d) 2D mapping of the local Ni valence state after the normalization process. Both scale bars in (a) and (d) are 5 μm.

Supplementary Tables

Supplementary Table 1 | Summary of the ICP-MS results: atomic ratio of the rod- and gravel-NMC.

	Atomic Ratio (%)		
Sample	Ni	Co	Mn
Rod_NMC	82.01	12.01	5.98
Gravel_NMC	79.60	10.18	10.22

Supplementary Table 2 | Summary of Ni K-edge absorption energies.

Sample name	Pristine state (eV)	First charged state (eV)
Rod-NMC hard XAS	8343.2	8344.4
Gravel-NMC hard XAS	8343.1	8344.5

Supplementary Table 3 | Summary of Ni L-edge $L3_{high}/L3_{low}$ ratios.

Sample name	Pristine state $L3_{high}/L3_{low}$	First charged state $L3_{high}/L3_{low}$
Rod-NMC TEY	0.2275	0.7657
Gravel-NMC TEY	0.2738	0.7355
Rod-NMC FY	0.5204	0.8319
Gravel-NMC FY	0.5882	0.8705

Supplementary Note 1:

We perform numerical simulation to understand the Li diffusion and stress evolution in the rod- and gravel-NMCs. Supplementary Figure 14 a and c show the finite element models of rod-NMC consisting of radially aligned grains and gravel-NMC consisting of randomly packed grains, respectively. In the simulation, the rod- and gravel-NMCs are delithiated (under charging) by a Li outflux imposed on the particle surface. The distributions of Li concentration and equivalent stress in the rod- and gravel-NMCs at different states of charge (SOCs) are shown in Supplementary Figure 14 b and d, respectively. In the rod-NMC, most Li diffuses outward along the aligned a-axis with the highest Li diffusivity. This continuous and less tortuous pathway for the Li outflux reduces the Li concentration gradient and thus improves the homogeneity of Li distribution in the rod-NMC (Supplementary Figure 14 b). From the mechanical aspects, the large anisotropic strain associated with the delithiation of nickel-rich NMC materials, especially in the deep SOC with a deleterious phase transition, generates a field of mechanical stress in NMC particles. For the rod-NMC, radially aligned grains can partially absorb this anisotropic strain through eliminating the mismatch strain between grains. In this scenario, the stress in the rod-NMC increases slowly and homogeneously during the delithiation, benefiting to the mechanical integrity of NMC particles (Supplementary Figure 14 b). For the gravel-NMC with randomly packed grains, the major Li diffusion pathway (i.e. along a-axis) is discontinuous and tortuous, which dramatically increases the heterogeneity of Li distribution within the particle (Supplementary Figure 14 d). More importantly, the randomly packed grains induce a large mismatch strain between grains, promoting the high stress concentrated at the grain boundaries (Supplementary Figure 14 d). This high concentrated stress (>2 GPa) is hardly sustainable for the weak grain boundaries and thus potentially triggers the local defects or cracks that further contribute to the electrochemical heterogeneity[1–3]."

Supplementary References

1. Mao, Y. *et al.* High-Voltage Charging-Induced Strain, Heterogeneity, and Micro-Cracks in Secondary Particles of a Nickel-Rich Layered Cathode Material. *Adv. Funct. Mater.* **29**, 1–11 (2019).
2. Yang, Y. *et al.* Quantification of Heterogeneous Degradation in Li-Ion Batteries. *Adv. Energy Mater.* **9**, 1900674 (2019).
3. Xia, S. *et al.* Chemomechanical Breakdown of Layered Cathode Materials undergoing Fast Charging in Lithium Batteries. *Nano Energy*. 53, 753-762, (2018)
